# A split Bregman method solving optimal reactive power dispatch for a doubly-fed induction generator-based wind farm

**DOI:** 10.1038/s41598-022-17761-4

**Published:** 2022-11-10

**Authors:** Fei Rong, Lingqi He, Sheng Huang, Mingcheng Lyu, Chao He, Xueping Li, Chunyi Zhao

**Affiliations:** 1 College of Electrical and Information Engineering, Hunan University, Changsha, 410082 China; 2 College of Automation Engineering, Nanjing University of Aeronautics and Astronautics, Nanjing, 211106 China; 3 Hunan Shuangpai Hydropower Co., Ltd, Shuangpai, 425200 China

**Keywords:** Energy science and technology, Engineering

## Abstract

This paper proposes an optimal reactive power control method to maximize wind farm revenue and minimize the total electrical losses of a doubly-fed induction generator (DFIG)-based wind farm. Specifically, the split Bregman method is used to solve the optimal control problem in a distributed manner. That is, the optimization problem is decomposed into sub-problems by the optimal distributed control strategy, and each sub-problem is solved independently in each local controller through the parallel method, which reduces the calculating burden and improves the information privacy. Thus, when a fault occurs, the proposed distributed control strategy can overcome the system fault and improve the reliability and security of the system. Furthermore, an economic financial model of annual revenue is contributed to examine the income impact with or without certified emission reduction (CER) by the clean development mechanism (CDM). Compared with the dual ascent (DA) method, sequential quadratic programming (SQP) method and the proportional dispatch method (PDM), the annual revenue (AR) of the wind farm using the proposed split Bregman method is the highest. Simulation results demonstrate that this method has promising performance in both optimization quality and computational efficiency.

## Introduction

It is undisputed that the optimal reactive power dispatch (ORPD) problem is one of focus areas in the research of power system research. The ORPD problem can be found in many real-world power system applications. For example, wind farms with large power generation capacity are expected to provide necessary reactive power support capability^1^. Generally, wind generation units are required with a certain amount of reactive output capability, and the reactive power output range should cover at least −0.3 to + 0.3 p.u., under rated real power output^2^. Having been developed for more than two decades, many classical optimization methods have been proposed to solve the ORPD problem, such as sequential quadratic programming (SQP)^3^, linear programming (LP)^4,5^, non-linear programming^6^ and interior point methods (IPM)^7^.

Despite the success in terms of the accuracy and robustness of classical methods on some specific problems, most of these methods have difficulty in dealing with the problems that have non-linear and discontinuous objectives^8^. To alleviate these drawbacks, some studies establish robust intelligent optimization models by introducing heuristic search strategies. In^9^, a hybrid solution strategy containing biogeography and predator–prey optimization technologies is proposed to handle multi-objective ORPD problems, which can significantly improve computational efficiency. Similarly^10^, presents a novel fuzzy adaptive heterogeneous comprehensive-learning particle swarm optimization (FAHCLPSO) algorithm with enhanced exploration and exploitation processes. In this method, a fuzzy system based on IF/THEN type fuzzy rules is designed to dynamically adopt and adjust the inertia weight when the proposed algorithm is running. Inspired by the ant hunting mechanism^11^, proposes a lion optimization (ALO) method, which takes the minimization of total real power losses into account and improves the voltage stability index on a series of IEEE node systems. To enhance global search ability, Mugemanyi et al. recommend a chaotic bat algorithm (CBA) to solve the ORPD problems^12^. In their method, the CBA results from introducing the chaotic sequences into the standard bat algorithm, which can effectively avoid the solutions trapping into the local optimum.

The above heuristic-based optimization methods have the same characteristic, they all adopt the control strategy of centralized control to optimize the ORPD problems. In this centralized control strategy, each terminal collects information and uploads it to the energy management system of the wind farm. However, the traditional centralized optimal scheduling may greatly increase the demand for hardware such as computers and storage devices. And the demand for communication broadband also increases dramatically. Furthermore, it greatly decreases the computational efficiency but increases the economic cost, which is completely incompatible with the requirements of the wind farm. It is worth mentioning that another disadvantage of the centralized optimal control strategies is that the optimization objective is not flexible. With the increase of the permeability of wind power, a large wind farm can be divided into small wind farms in different areas, optimal scheduling objectives for different areas may be different, the unified optimization objection for the different areas may not be suitable and it exists reliability problem. If the centralized optimal dispatching center is attacked, the dispatching of the whole wind farm may collapse with serious consequences. Therefore, the centralized optimal scheduling model cannot adapt to the future development of wind farms.

Being different from the centralized control, the distributed optimal scheduling can decompose the large-scale wind farm scheduling problem into several small-scale sub-problems. Therefore, the overall optimization can be achieved through the independent optimization calculation and coordination of interactive information. Moreover, it allows information processing under independent decision-making of the transmission system operator and distribution system operator, and coordinates the whole distribution systems by boundary information exchange^13^. Due to the fact that the abovementioned advantages of the distributed or decentralized systems, various distributed or decentralized methods are proposed to address the above issues. As one of dual-based methods, dual ascent (DA) is widely used to develop distributed optimization and control algorithms for power systems^14^. However, a known drawback of DA with conventional gradient methods is that it often suffers from slow convergence and its heavy dependence on parameters. The optimality condition decomposition (OCD) method is used for solving the optimal power flow problem^15^. The accuracy of the OCD decomposition technique has been evaluated and compared with the conventional continuation power flow framework and sequential quadratic programming techniques. By comparing the preliminary approximation with the Monte Carlo method, it is determined that the proposed technique provides an acceptable preliminary approximation of the worst-case, average-case and best-case scenarios of the available transfer capability subjected to wind power fluctuation. However, this method has low computational efficiency. In the OCD technique, primal and dual variables are assigned to a specific sub-problem. During an interior point iteration, all foreign variables are fixed to their previous value. By penalizing the coupling variables in the objective, convergence is achieved under certain assumptions (e.g. relatively weakly coupled sub-problems). Those assumptions cannot be guaranteed for any problem and the convergence conditions of OCD are usually hard to check before solving the optimization problem^16^. In a different way from OCD, the auxiliary problem principle (APP) algorithm based on the augmented Lagrangian method is introduced to implement the distributed power flow^17^. The proposed approach in this paper enables multiple interconnected systems with their own sub-objectives to share their resources and participate in an electricity market without implicitly sharing information about their local generators or internal network parameters. In this way, the coupled large-system optimization problem is decoupled into independent sub-optimization problems, and the average number of iterations can be reduced. As an improvement of the APP, the alternating direction method of multiplier (ADMM) gives a sequential update on internal and external variables, which reduces necessary information exchange and leads to communication between neighboring regions only^16^. An ADMMs based method is presented to achieve global optimality of power flows in power-distribution systems^18^. Appropriate linear approximations to the power flow equations are utilized in order to facilitate computationally affordable ADMM-based controller design. The ADMM algorithm proposed in this paper has the advantage of being easy to expand. However, it requires making assumptions about the mismatch between the commanded set point and the current system output, which results in the disadvantage of affecting the accuracy of the algorithm. In a different attempt^19^, develops a distributed sequential coordination scheme based on the analytical target cascading (ATC) method to solve the optimum power flow problem. The proposed parallelized coordination strategy significantly reduces the computational complexity of the developed formulation and makes it favorable in large-scale networks. However, ATC has different mathematical expressions according to the expression of the penalty term, and its solution efficiency is also different. The selection of an inappropriate penalty expression will reduce its efficiency.

According to the above analysis, the approaches to solve the optimal power flow problem presented in the literature can be categorized into three classes: centralized solution algorithms, decentralized and distributed solution algorithms. Although the decentralized control strategy can eliminate the requirement of the central unit, it is difficult to obtain the optimal solution^20^. To address the above issues, a distributed ORPD strategy using the split Bregman method (SBM) is proposed in this paper, aiming to maximize wind farm revenue and minimize the total electrical losses inside the wind farm. SBM is one of the popular methods, which has been used for optical flow estimation^21^, image inpainting^22^, image reconstruction^23^, image denoising^24^, etc. As far as we know, the SBM algorithm has not been used in the field of power optimization. Compared to other optimization methods, such as the APP, the OCD, and the ATC, the SBM has the following advantages:

1. The SBM introduces auxiliary variable *b*, which increases the fault tolerance rate of the algorithm.
2. Compared with the continuation methods, the value of the penalty factor in SBM remains constant. Choosing an appropriate value for the penalty factor can minimize the condition number of the sub-problems, resulting in fast convergence for iterative optimization methods and easily parallelizable.
3. It also has a relatively small memory footprint compared with other methods and can also avoid the problem of numerical instabilities^24^.

In this paper, the reactive power dispatch between the wind turbines is integrated with the optimal reactive power control strategy. The optimal splitting strategy of the rotor side converter and the grid side converter is used to control the reactive power of the wind turbines, which can achieve high computational efficiency, flexible selection of optimization objectives, and strong reliability. What is more, by extending the distributed architecture to the redundant system, the availability of the system is improved. Through modularizing the system, the reusability of modules can be improved and the expansibility of the system is also higher.

The main contributions of this paper are summarized as follows.

1. A financial model of annual revenue to examine the impact with/without certified emission reduction (CER) income by the clean development mechanism (CDM) is conducted.
2. A distributed ORPD strategy is designed for the wind farm. Compared to the centralized optimal control, the requirement of a central controller is eliminated, which can achieve high computational efficiency, flexible selection of optimization objectives, strong reliability and easily parallelizable.
3. A split Bregman method is utilized to complete the process, which has a relatively small memory footprint and can avoid the problem of numerical instabilities. Compared to the DA method, the split Bregman method has less dependence on parameters, so it has better robustness and scalability.

This paper is organized as follows: the introduction of the split Bregman method is presented in “Split Bregman method” section, the loss models of wind turbines is presented in “Loss models of wind turbines” section, the optimal distributed reactive power control strategy based on the split Bregman method is described in “Distributed ORPD strategy with the split Bregman method” section, the case study compared the proposed distributed control strategy with the traditional centralized control strategy is analysed in “Case study” section, and the conclusion is drawn in “Conclusion” section.

### Split Bregman method

The split Bregman method motivated by Bregman distance has been shown its usefulness for solving L1 and L1-liked problems. In this subsection, we will briefly introduce split Bregman iteration. Split Bregman method is proposed by the Goldstein and Osher^25^ to solve the general optimization problem as follows.

1 $$\mathop {\min }\limits_{u} H(u) + \left\| {\varphi (u)} \right\|_{1}$$

2 $${\text{s}}.{\text{t}}.\quad \varphi (u{) = }d$$

where, ${||.||}_{1}$ denotes the L1 norm, and s.t. denotes the constraint objective, both *H*(*u*) and ${\Vert \varphi (u)\Vert }_{1}$ are convex functions. The control parameter λ is a positive constant. In the split Bregman iteration, ${\Vert \varphi (u)\Vert }_{1}$ is firstly replaced by ${\Vert d\Vert }_{1}$. Then the corresponding variational model with split Bregman method is transformed as follows^24^.

3 $$(u^{(k + 1)} ,d^{(k + 1)} ) = \mathop {\arg \min }\limits_{u, \, d} H(u) + \left\| d \right\|_{1} + \frac{\lambda }{2}\left\| {d^{(k)} - \varphi (u) - b^{(k)} } \right\|_{2}^{2}$$

The iteration steps of the split Bregman algorithm are shown in Table 1. First, initialize parameters. Second, *u* is iterated, taking* u* as an independent variable and other variables as constants. Then, *d* is iterated, taking* d* as independent variable and other variables as constants. Finally, the intermediate variable *b* is iterated until the convergence condition is satisfied.

**Table 1:** The split Bregman algorithm.

Split Bregman iteration
1: Initialization: *u*^(0)^ = 0, *d*^(0)^ = 0, *b*^(0)^ = 0
2: for *k* = 0 to *N* − 1
Step 1: $$u^{(k + 1)} = \mathop {\min }\limits_{u} H(u) + \frac{\lambda }{2}\left\| {d^{(k)} - \varphi (u) - b^{(k)} } \right\|_{2}^{2}$$
Step 2: $$d^{(k + 1)} = \mathop {\min }\limits_{d} \left\| d \right\|_{1} + \frac{\lambda }{2}\left\| {d - \varphi (u^{(k + 1)} ) - b^{(k)} } \right\|_{2}^{2}$$
Step 3: $$b^{(k + 1)} = b^{(k)} + (\varphi (u^{(k + 1)} ) - d^{(k + 1)} )$$
3: end
4: output:$$u^{(N)}$$

According to the split Bregman method^24^, the iteration variable *b* is initialized with zero and updated by

4 $$b^{(k + 1)} = b^{(k)} + (\varphi (u^{(k + 1)} ) - d^{(k + 1)} )$$

### Loss models of wind turbines

The power losses of a wind turbines in this paper include copper losses in the DFIG, losses in the converters and the filter^26^.

The steady-state voltage equation of DFIG working in stator voltage-oriented coordinate system is as follows^27^:

5 $$\left[\begin{array}{l}{V}_{s}\\ 0\\ {V}_{rd}\\ {V}_{rq}\end{array}\right]=\left[\begin{array}{llll}{R}_{s} & {-X}_{s}& 0 & {-X}_{m}\\ {X}_{s}& {R}_{s}& { X}_{m} &0\\ { 0 }&{-sX}_{m}& {R}_{r}& {-sX}_{r}\\ {sX}_{m} & 0& {sX}_{r}& {R}_{r}\end{array}\right] \left[\begin{array}{l}{I}_{sd}\\ {I}_{sq}\\ {I}_{rd}\\ {I}_{rq}\end{array}\right]$$

where, stator inductance ${X}_{s}$ equals ${X}_{ls}+{X}_{m}$, rotor inductance ${X}_{r}$ equals ${X}_{lr}+{X}_{m}$,${X}_{ls}$ is stator leakage inductance and ${X}_{lr}$ is rotor leakage inductance,${X}_{m}$ is mutual inductance.${V}_{s}$ and ${R}_{s}$ are stator voltage and stator resistance, respectively.${V}_{rd}$ and ${V}_{rq}$ are components of rotor voltage in d-axis and q-axis, respectively.${R}_{r}$ is rotor resistance and s is rotor slip.

The rotor d-axis and stator q-axis current can be calculated as:

6 $${I}_{rd}=-\frac{u{X}_{s}{w}_{s}}{{V}_{s}{X}_{m}{w}_{r}}{P}_{mec}$$

7 $${I}_{sq}=\frac{{Q}_{s}}{{V}_{s}}$$

The current of rotor q-axis and stator d-axis can be calculated as:

8 $${I}_{rq}=-\frac{u{Q}_{s}\left({X}_{s}^{2}+{R}_{s}^{2}\right)}{{X}_{s}{X}_{m}{V}_{s}}+\frac{u{R}_{s}{w}_{s}}{{X}_{m}{V}_{s}{w}_{r}}{P}_{mec}-\frac{u{V}_{s}}{{X}_{m}}$$

9 $${I}_{sd}=\frac{{R}_{s}}{{X}_{s}{V}_{s}}{Q}_{s}+\frac{{w}_{s}}{{V}_{s}{w}_{r}}{P}_{mec}$$

where ${w}_{s}$ is the angular frequency of the stator windings, ${w}_{r}$ is the angular frequency of the rotor windings. ${P}_{mec}$ is the mechanical power output of the wind power generator and ${Q}_{s}$ is the reactive power of the stator. *u* is the turns ratio between stator and rotor.

The total copper losses in the DFIG can be calculated as:

10 $${P}_{loss,cu}={R}_{s}\left({I}_{sd}^{2}+{I}_{sq}^{2}\right)+{R}_{r}({I}_{rd}^{2}+{I}_{rq}^{2})$$

The losses of the converter can be divided into the machine-side and the grid-side, according to^28^, the total losses in converter can be expressed as:

11 $${P}_{loss,converter}=(({V}_{IGBT}+{V}_{sw,T}+{V}_{sw,D})\frac{6\sqrt{2}}{\pi }{I}_{rms}+3{r}_{IGBT}{I}_{rms}^{2})$$

where ${I}_{rms}$ is the root mean square value of the current,${V}_{sw,T}$ and ${V}_{sw,D}$ are two voltage drops.${f}_{sw}$ is the switching frequency.${V}_{IGBT}$ is the voltage across the collector and emitter of the IGBT,${r}_{IGBT}$ is the lead resistance of the IGBT. For the grid-side and the machine-side respectively, the current can be calculated as:

12 $${I}_{rms}^{RSC}=\sqrt{{I}_{rd}^{2}+{I}_{rq}^{2}}$$

13 $${I}_{rms}^{GSC}=\sqrt{{I}_{gd}^{2}+{I}_{gq}^{2}}$$

The grid side converter currents ${I}_{gd}$ and ${I}_{gq}$ can calculated as follows:

14 $${I}_{gd}=\frac{{I}_{rd}{V}_{rd}+{I}_{rq}{V}_{rq}}{{V}_{s}}$$

15 $${I}_{gq}=\frac{{Q}_{g}}{{V}_{s}}$$

16 $${Q}_{g}+{Q}_{s}={Q}_{ref}$$

Hence, the loss in the grid side filter can be calculated according Eqs. (14) and (15):

17 $${P}_{loss,filter}={R}_{filter}({I}_{gd}^{2}+{I}_{gq}^{2})$$

Where ${Q}_{g}$ is the reactive power provided by the grid side converter and ${Q}_{ref}$ is the total reference reactive power. ${R}_{filter}$ is the filter resistance. So the total losses of the DFIG can be calculated as:

18 $${P}_{loss,DFIG}={P}_{loss,cu}+{P}_{loss,converter}+{P}_{loss,filter}$$

## Distributed ORPD strategy with the split Bregman method

Split Bregman method has the advantage of fast convergence for the iterative optimization methods such as Newton and Gauss–Seidel, which makes extensive use of Gauss–Seidel and Fourier transform, hence it is easy to be parallelized^24^. It allows each of nodes in the network to have its own local objective function and a local constrains. Each node solves the local optimization problem for the global variables and all local variables can be updated in parallel iterations. The problem can be solved with all local variable eventually converging to the global optimal value and the numerical instabilities can be avoided.

**Figure 1:**
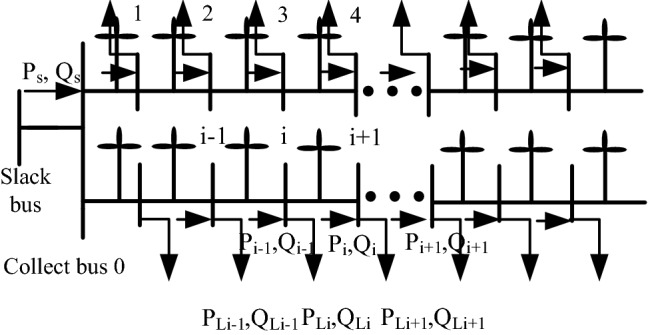
Schematic diagram of the wind farm network.

To illustrate the problem of reactive power optimization in wind farms, consider the wind farm network in Fig. 1.

The main factors affecting the profit of wind farm are initial capital costs (ICC), annual costs of operation and maintenance (AOM), annual wind electricity production (AEP) and carbon reduction (CR) income^29^. The objective function is formulated to maximize the annual revenue (AR) of the wind farm. The overall objective function can be expressed as follow:

19 $$AR = Ce*AEP - AOM - FCR*ICC + CR$$

ICC represent initial capital costs, the total initial investment cost in this paper is 108.94 million USD. AOM represent annual operation and maintenance, the total AOM in this paper is 1.657527 million USD.

Ce is the electricity selling price and based on China’s national conditions, the value here is 0.0871 dollar per kWh. FCR is the fixed charge gate, is a factor by which the ICC is multiplied to convert the initial capital cost into an annual cost, the value here is 0.1158^30^.

CR represent the revenue of the clean development mechanism (CDM). The core content of the CDM mechanism is that developed countries cooperate with developing countries by providing funds and technologies to implement projects with greenhouse gases emission reduction effects in developing countries, and the greenhouse gases emission reductions produced by the projects are used by developed countries to implement Kyoto Agreement^31^. Please noted that CR is equal to zero when the wind farms is not successfully registered the CDM project. The calculation formula of CR is as follows:

20 $$CR = AEP*PC*EF$$

PC is the carbon equivalent transaction price which is set at 16.4$/t in this paper^32^, EF represent the emission factors are calculated via weighted both marginal emission factor (MEF) and capacity emission factor (CEF):

21 $$EF = W_{MEF} *MEF + W_{CEF} *CEF$$

where $W_{MEF}$ and $W_{CEF}$ is set as 0.5, based on China’s national conditions, the values of the MEF and CEF are set as 0.9489 and 0.3157 respectively^32^.

AEP represent annual energy production*,* the calculation formula of AEP is as follows:

22 $$AEP = \sum\limits_{t = 1}^{T} {\left[ {\left( {\sum\limits_{k = 1}^{n} {P_{wt,k} (t) - P_{loss,windfarm} (t)} } \right)*\frac{8760}{T}} \right]}$$

Here, $P_{wt,k} (t)$ is the active power of WT *k* at time *t*, *n* is the number of wind turbines, $P_{loss,windfarm} (t)$ is the total power loss of the wind farm at time *t*, and *T* is the total number of sampling points^33^. The active power output of the DFIG at each control period can be considered constant, the wind farm operator generates the optimal reactive power references for the DFIG stator and grid side controller to minimize the total power losses inside the wind farm. Hence, $P_{loss,windfarm}$ use the split Bregman method calculations as follows:

23 $$\begin{aligned} & \quad \quad \quad \quad \quad \quad \quad \quad \quad {{\varvec{\zeta}}}^{{\varvec{S}}{\varvec{p}}{\varvec{l}}{\varvec{i}}{\varvec{t}}\boldsymbol{ }{\varvec{B}}{\varvec{r}}{\varvec{e}}{\varvec{g}}{\varvec{m}}{\varvec{a}}{\varvec{n}}} \\ & P_{loss,windfarm}={r}_{0}\frac{({{Q}_{0}^{-})}^{2}}{{V}_{s}^{2}}+\frac{\lambda }{2}{({Q}_{0}^{+}-{Q}_{0}-{ b}_{0}^{{Q}^{+}})}^{2}+\frac{\lambda }{2}{({Q}_{0}^{-}-{Q}_{refwf}-{b}_{0}^{{Q}^{-}})}^{2}+\frac{\lambda }{2}{({U}_{0}^{+}-{U}_{0}-{b}_{0}^{{U}^{+}})}^{2}+\frac{\lambda }{2}{({U}_{0}^{-}-{U}_{s}-{b}_{0}^{{U}^{-}})}^{2})+\sum_{i=1}^{n}({r}_{i}\frac{({{Q}_{i}^{-})}^{2}}{{V}_{s}^{2}}+{P}_{loss,DFIG}+\frac{\lambda }{2}{({Q}_{i}^{+}-{Q}_{i}-{ b}_{i}^{{Q}^{+}})}^{2}+\frac{\lambda }{2}{({Q}_{i}^{-}-{Q}_{i-1}-{b}_{i}^{{Q}^{-}})}^{2}+\frac{\lambda }{2}{({U}_{i}^{+}-{U}_{i}-{b}_{i}^{{U}^{+}})}^{2}+\frac{\lambda }{2}{({U}_{i}^{-}-{U}_{i-1}-{b}_{i}^{{U}^{-}})}^{2})\\ &\quad \quad \quad \quad \quad \quad \quad \quad \quad \quad \quad \quad \quad \quad \quad \quad {s.t.}\end{aligned}$$

24 $${Q}_{i}^{+}-{Q}_{i}^{-}={Q}_{i}^{s}+{Q}_{i}^{g}$$

25 $${Q}_{i}^{+}={Q}_{i},{Q}_{i}^{-}={Q}_{i-1},{Q}_{0}^{+}={Q}_{0}, {Q}_{0}^{-}={Q}_{refwf}, {Q}_{n}=0$$

26 $${U}_{i}^{+}={U}_{i}^{-}-2\left({r}_{i}{P}_{i}+{x}_{i}{Q}_{i}^{-}\right)$$

27 $${V}_{s}^{2}({\epsilon }^{2}-2\epsilon )\le {U}_{i}^{+}\le {V}_{s}^{2}({\epsilon }^{2}+2\epsilon )$$

28 $${U}_{i}^{+}={U}_{i},{U}_{i}^{-}={U}_{i-1},{U}_{0}^{+}={U}_{0},{U}_{0}^{-}=0$$

The power flow in the wind farm network expressed by a set of recursive equations is called *DistFlow branch equations*^34^. Here, the voltage constraints conform the ANSI C84.1-2006 standard. Under normal operations, the changes in voltage from node to node are smaller compared to the loss of real and reactive power flows themselves, thus we have assumed ${V}_{i}^{2}\approx {V}_{s}^{2}$, ${V}_{s}$ represents the voltage of the slack bus and ${U}_{i}$=${V}_{i}^{2}$-${V}_{s}^{2}$. ${r}_{i}$ and ${x}_{i}$ are the resistance and inductance of the cable, *Q*_*refwf*_ is the total reference reactive power of the wind farm.

It is assumed that ${Q}_{i}^{+}and\,{U}_{i}^{+}$ represent local variable ${Q}_{i} ,{U}_{i}$ respectively. The *i* represents the *i*th feeder, for all *i* $\in n,$ *n *is the number of buses. ${Q}_{i}^{-}$, ${U}_{i}^{-}$ represent local variable ${Q}_{i-1}$ , ${U}_{i-1}$ respectively, which means that the energy is transferred between nodes. We note that the ${P}_{i}$ are auxiliary constant parameters and can be measured by each controller. The reactive optimization problem can be formulated as a consistency problem.${Q}_{i}^{s} and {Q}_{i}^{g}$ represent reactive power of DFIG stator side and grid side, respectively.

The quadratic terms in the objective function with $\frac{\lambda }{2}$ penalty factor represent local variables penalties are different from the global variables, but they do not change the optimal value and require the local and global variable are equal at the optimum. *b* is an intermediate variable that minimizes both ${Q}^{+}$ and ${Q}^{-}.$ The algorithm can minimize the function value in such a way that ${Q}^{+}$ and ${Q}^{-}$ will always keep with the feasible set and satisfy the constraints. The steps of the algorithm are as follows:

Minimization step: the minimization step including the equality and inequality constraints, which can be solved analytically. To simplify the expression, each node can perform the minimization step independently.

Global variables update step: the global variables *Q *and *U* is update in this step. This requires that each local variable in each node can calculate the new value for ${Q}_{i}$, ${Q}_{i+1}$ and communicate with the neighboring nodes. The new value is the average value of the respective local variables. The update step as follow:

29 $${Q}_{i}(k+1)=\frac{1}{2}\left({Q}_{i}^{+}(k+1)+{Q}_{i+1}^{-}(k+1)\right)$$

30 $${U}_{i}(k+1)=\frac{1}{2}\left({U}_{i}^{+}(k+1)+{U}_{i+1}^{-}(k+1)\right)$$

31 $${Q}_{n}(k+1)=0,{Q}_{0}(k+1)={Q}_{1}^{-}(k+1),{U}_{n}={U}_{n}^{+}$$

Intermediate variable iteration update step: iterate the intermediate variables with the optimal value of each local variable, ensure that the iterative value of the intermediate variables is the optimal value. The update step as follow:

32 $${b}_{i}^{{Q}^{+}}\left(k+1\right)= {b}_{i}^{{Q}^{+}}\left(k+1\right)+({Q}_{i}^{+}\left(k+1\right)-{Q}_{i}(k+1))$$

33 $${b}_{i}^{{Q}^{-}}\left(k+1\right)= {b}_{i}^{{Q}^{-}}\left(k+1\right)+( {Q}_{i}^{-}\left(k+1\right)-{Q}_{i-1}(k+1))$$

34 $${b}_{i}^{{U}^{+}}\left(k+1\right)= {b}_{i}^{{U}^{+}}\left(k+1\right)+( {U}_{i}^{+}\left(k+1\right)-{U}_{i}\left(k+1)\right)$$

35 $${b}_{i}^{{U}^{-}}\left(k+1\right)= {b}_{i}^{{U}^{-}}\left(k+1\right)+( {U}_{i}^{-}\left(k+1\right)-{U}_{i-1}\left(k+1\right))$$

The split Bregman algorithm maintains the synchronous communication between all nodes, and the local variables communicate between adjacent nodes. Once the algorithm converges, the local variables will actually correspond to the optimal feasible solution and leads convergence of the global variables to optimized value.

## Convergence and stopping criteria

The convergence proof of the algorithm is presented in Supply. Appendix. If the stopping criteria are satisfied in every controller inside the wind farm, the converged optimal result is obtained. The primal residual for Eq. (3) is given by:

36 $$r^{k + 1} = d^{k + 1} - \varphi (u^{k + 1} )$$

And the dual residual is

37 $$s^{k + 1} = \varphi (u^{k + 1} ) - \varphi (u^{k} )$$

A suitable balancing conditions is when the residuals is smaller than some threshold, in this paper we use the primal residual smaller than a threshold as the stopping criteria.

38 $$\left\| {r^{k + 1} } \right\|_{2}^{2} \le \varepsilon^{pri}$$

where $\varepsilon^{pri}$ are tolerances for primal feasibility, the value of $\varepsilon^{pri}$ is set to $10^{ - 4}$ in this paper.

## Case study

A wind farm of 100 MW installed capacity which has 20 turbines was built to test the proposed strategy. Each wind turbine has an installed capacity of 5 MW. The gross properties for the DFIG are shown in Table 2^35^.

**Table 2:** The parameters of the DFIG.

Parameters	Value	Per unit value
Rated mechanical power	5.0 MW	0.05 p.u
Rated stator phase voltage	548.48 V (rms)	0.016 p.u
Rated Rotor phase voltage	381.05 V (rms)	0.011 p.u
Rated stator current	2578.4 A (rms)	1.47 p.u
Rated rotor current	3188.7 A (rms)	1.82 p.u
Rated stator frequency	50 Hz	1.0 p.u
Rated rotor speed	1170 rpm	1.0 p.u
Stator winding resistance	1.552 mΩ	0.00014 p.u
Rotor winding resistance	1.446 mΩ	0.00013 p.u
Stator leakage inductance	1.2721 mH	0.0367 p.u
rotor leakage inductance	1.1194 mH	0.0323 p.u
Magnetizing inductance	5.5182 mH	0.1591 p.u
Base mechanical power	100 MW	
Base voltage	33 kV	
Base current	1749.55 A	
Base flux linkage	1.7459 Wb	
Base impedance	10.89 Ω	

In this paper, the proposed split Bregman method is compared with the dual ascent (DA) method, sequential quadratic programming (SQP) method and the proportional dispatch method (PDM). All simulations are implemented using matlab language programming in MATLAB 2018 using a personal computer with Intel Core i3-8100U CPU, 2.60 GHz processor and 12 GB of RAM. The comparison algorithm is described as follows:

### Sequential quadratic programming

Sequential quadratic programming transforms complex constrained optimization problems into simple quadratic sequential problem solving, which was first proposed by Wilson in 1963, it has the advantages of strong global convergence and local superlinear convergence. The SQP method also maintains the local fast convergence rate of Newton's method

39 $$Min.f(x)$$

40 $$s.t.\left\{ \begin{gathered} h(x) = 0 \hfill \\ g(x) \le 0 \hfill \\ \end{gathered} \right.$$

Using Taylor expansion, the objective function of the constraint problem is simplified into a quadratic function at the iteration point $x^{k}$. After simplifying the constraint function into a linear function, the following quadratic programming problem is obtained

41 $$\begin{gathered} Min.f(x) = \frac{1}{2}(x - x^{k} )^{T} \nabla^{2} f(x^{k} )(x - x^{k} ) \hfill \\ \, + \nabla f(x^{k} )^{T} (x - x^{k} ) \hfill \\ \end{gathered}$$

42 $$s.t.\left\{ \begin{gathered} \nabla h(x^{k} )^{T} (x - x^{k} ) + h(x^{k} ) = 0 \hfill \\ \nabla g(x^{k} )^{T} (x - x^{k} ) + g(x^{k} ) = 0 \hfill \\ \end{gathered} \right.$$

### Proportional dispatch method

Considering the instantaneous power generation level of each generator, the proportional dispatch method distribute the reactive power in a proportional way as follows:

43 $${Q}_{iref}=\frac{{Q}_{imax}}{\sum_{i=1}^{n}{Q}_{imax}}{Q}_{totalref}$$

where ${Q}_{iref}$ is the reference value for reactive power of each wind generator and ${Q}_{imax}$ is the maximum of the reactive power that each generator can absorb/generate. The ${Q}_{totalref}$ is the total reactive power reference for the wind farm. The monitoring system distributes reactive power generation tasks among wind turbines according to the Eq. (43) so the reference value of reactive power ${Q}_{iref}$ of each wind turbine is obtained.

### Dual ascent method

Consider the equality-constrained optimization problem

44 $$Min.f(x)$$

45 $$s.t. \, h(x) = 0$$

Write the limiting conditions into the target through the Lagrange multiplier, and the Lagrange function is:

46 $$L(x,y) = f(x) + y^{T} (Ax - b)$$

The process of dual ascent method includes the following three steps, first, update decision variables as follows:

47 $$x^{k + 1} = \mathop {\arg \min }\limits_{x} L(x,y^{k} )$$

Then update the Lagrange multiplier:

48 $$y^{k + 1} = y^{k} + a^{k} (Ax^{k + 1} - b)$$

where $a$ is the update step of the Lagrange multiplier. Finally loop iterate decision variables and Lagrange multipliers until convergence condition is satisfied.

**Figure 2:**
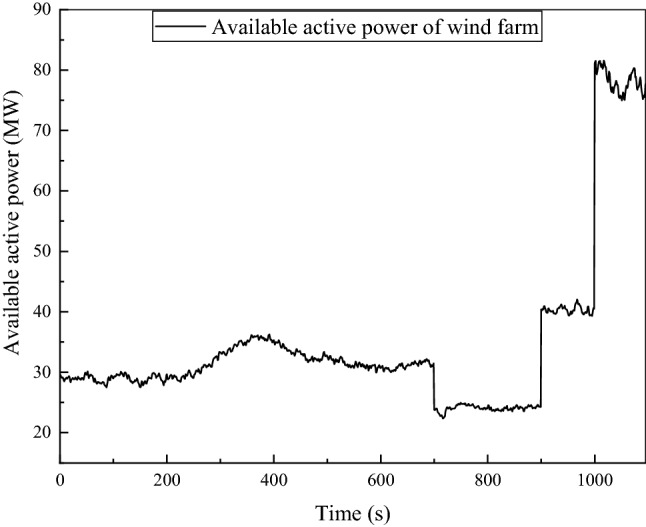
Available active power for the wind farm.

A wind data record, which is sampled every 8 h, totally 1095 data is used to be the wind input of the wind farm. Figure 2 shows the limit available active power command from the transmission system operator. From 0 to 700 s, the limit available active power fluctuates between 30 and 40 MW and decrease to 20 MW during 700 s to 900 s, gradually increase to 40 MW during 900 s to 1000 s. After 1000 s, the wind farm operate without power constraints.

**Figure 3:**
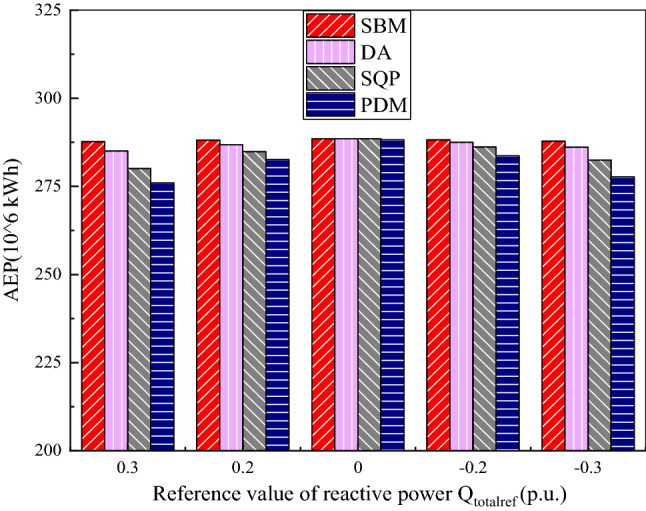
The value of annual electricity production.

**Figure 4:**
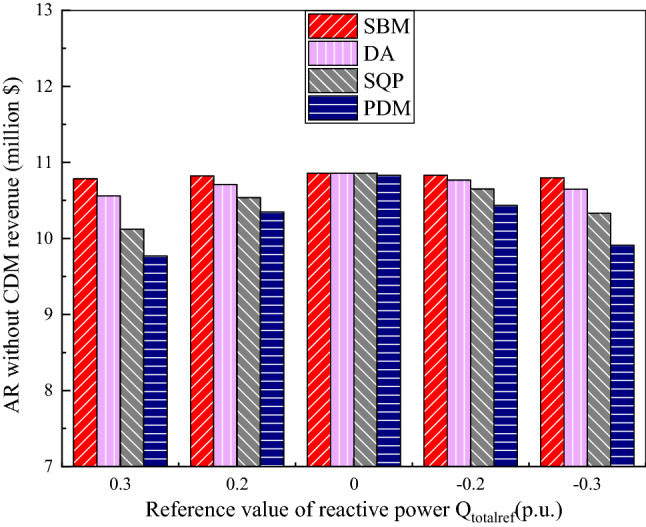
AR without CDM revenue.

**Figure 5:**
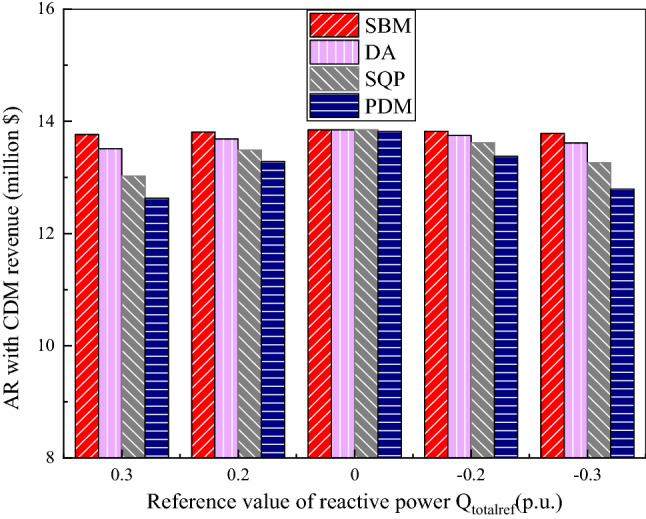
AR with CDM revenue.

**Figure 6:**
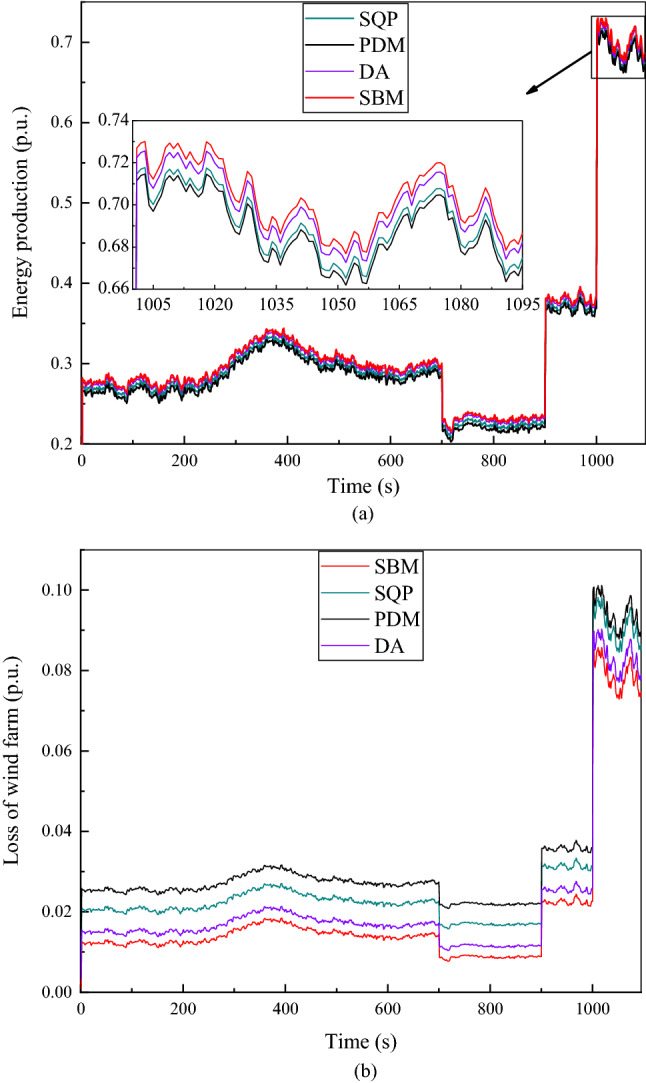
The given value of reactive power ${Q}_{totalref}$ is 0.3 p.u. (**a**) Energy production at continuous time points. (**b**) Loss of wind farm at continuous time points.

**Figure 7:**
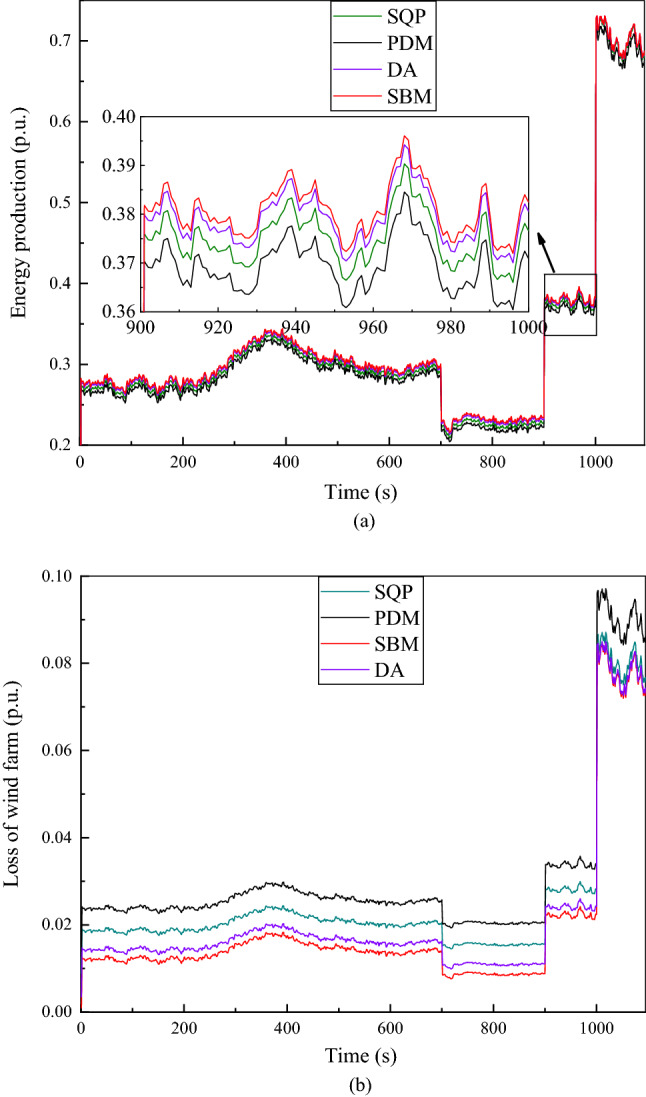
The given value of reactive power ${Q}_{totalref}$ is −0.3 p.u. (**a**) Energy Production at continuous time points. (**b**) Loss of wind farm at continuous time points.

**Figure 8:**
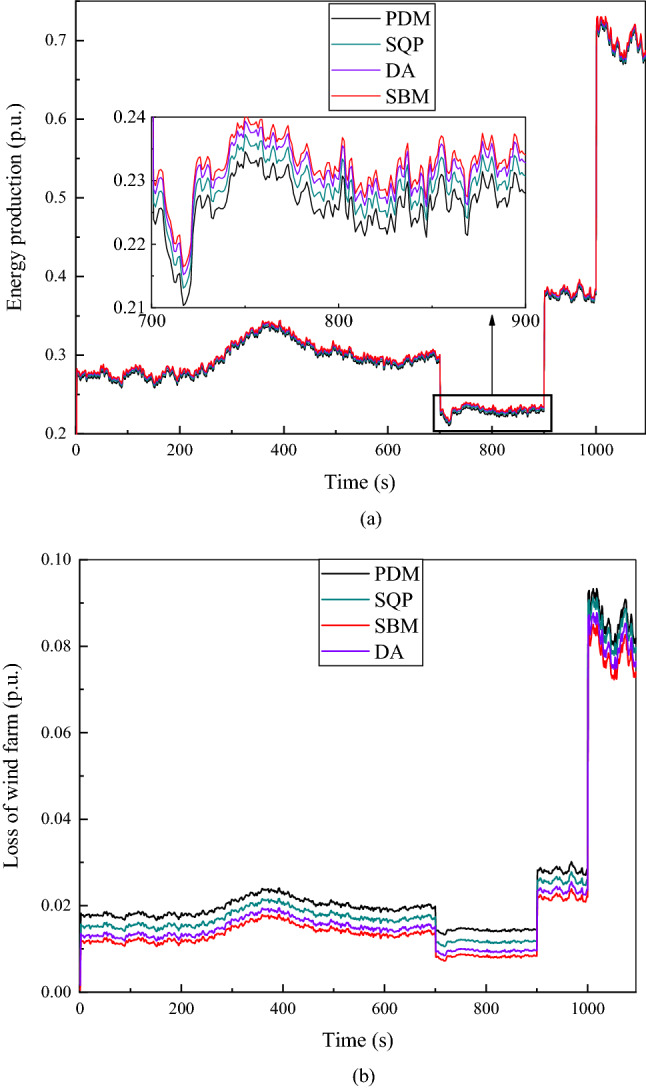
The given value of reactive power ${Q}_{totalref}$ is 0.2 p.u. (**a**) Energy production at continuous time points. (**b**) Loss of wind farm at continuous time points.

**Figure 9:**
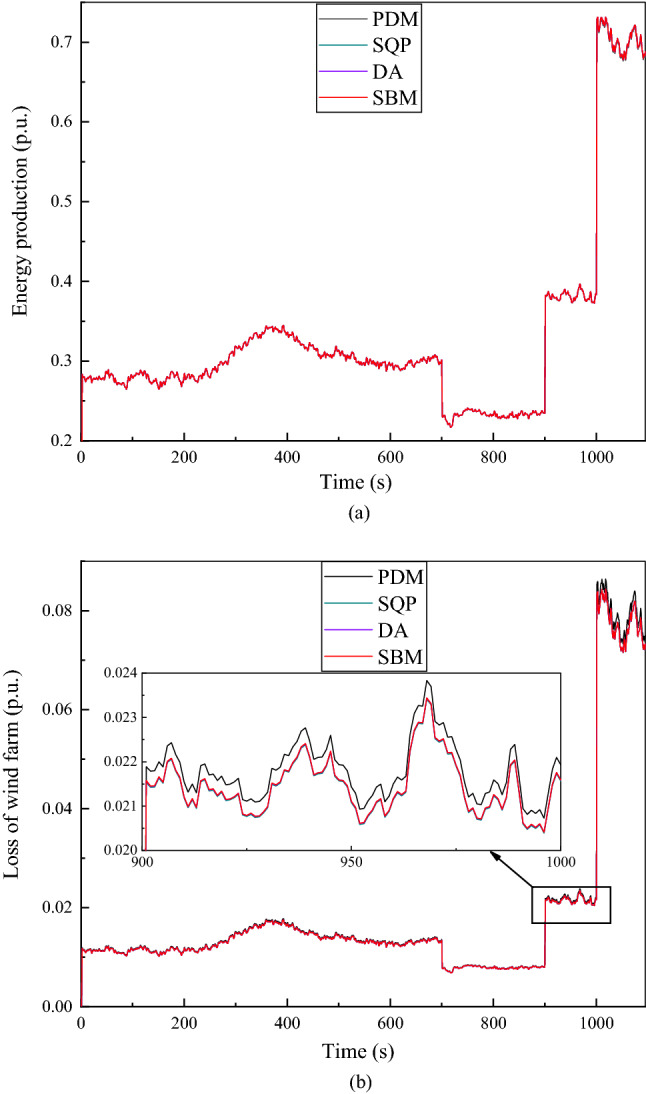
The given value of reactive power ${Q}_{totalref}$ is 0 p.u. (**a**) Energy production at continuous time points. (**b**) Loss of wind farm at continuous time points.

**Figure 10:**
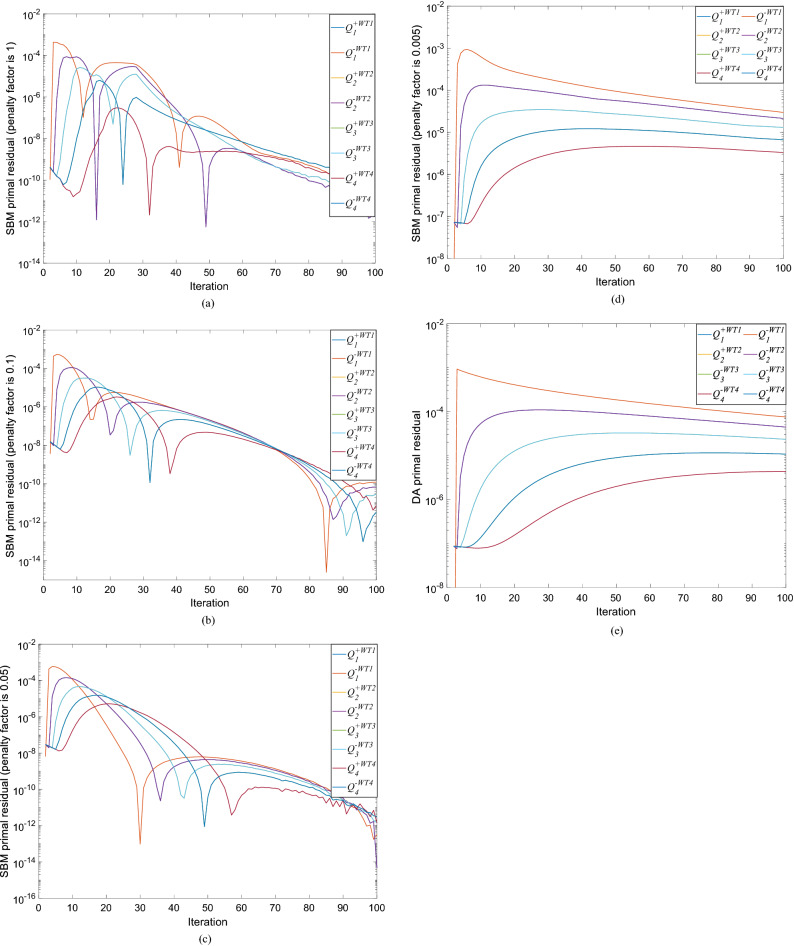
The given value of reactive power ${Q}_{totalref}$ is − 0.2 p.u. (**a**) SBM primal residual convergence diagram with penalty factor 1. (**b**) SBM primal residual convergence diagram with penalty factor 0.1. (**c**) SBM primal residual convergence diagram with penalty factor 0.05. (**d**) SBM primal residual convergence diagram with penalty factor 0.005. (**e**) DA primal residual convergence diagram.

The annual revenue (AR) of wind farm, energy production and total loss of wind farm calculated by SBM which is compared with the DA, SQP and the PDM are shown in the Figs. 3, 4, 5, 6, 7, 8, 9, and 10. The range of reactive power reference values for the wind farm calculated in this paper is −0.3 p.u. to 0.3 p.u. Table 3 show the annual revenue of wind farm with or without the CDM revenue calculated by different algorithm. The value of annual electricity production is shown in Fig. 3, it can be seen from the figure that when the given reactive power value is plus and minus 0.3 p.u, the value of AEP is relatively small. The AEP calculated by the SBM algorithm is 287.67 million kWh and 287.84 million kWh, respectively. The AEP calculated by DA is 285.08 million kWh and 286.12 million kWh, respectively. The AEP calculated by SQP is 280.07 million kWh and 282.50 million kWh, respectively. The AEP calculated by PDM is 276.01 million kWh and 277.65 million kWh, respectively. Compared with the DA algorithm, the AEP calculated by the SBM algorithm increased by 0.91% and 0.60%. Compared with the SQP algorithm, the AEP calculated by the SBM algorithm increased by 2.71% and 1.89%. Compared with the PDM algorithm, the AEP calculated by the SBM algorithm increased by 4.22% and 3.67%. When the given value of reactive power is 0 p.u, the value of AEP is relatively large, about 288 million kWh.

**Table 3:** The value of AR.

Q_totalref_ (p.u)	SBM without CDM revenue ($)	SBM with CDM revenue ($)	DA without CDM revenue ($)	DA with CDM revenue ($)	SQP without CDM revenue ($)	SQP with CDM revenue ($)	PDM without CDM revenue ($)	PDM with CDM revenue ($)
0.3	10,783,278	13,766,335	10,557,689	13,513,888	10,121,318	13,025,565	9,767,692	12,629,838
0.2	10,822,473	13,810,196	10,709,243	13,683,486	10,535,914	13,489,521	10,346,907	13,278,012
0	10,855,571	13,847,235	10,855,519	13,847,224	10,855,222	13,846,844	10,831,183	13,819,943
−0.2	10,831,183	13,819,943	10,766,729	13,747,816	10,650,015	13,617,206	10,434,007	13,375,481
−0.3	10,798,085	13,782,905	10,648,273	13,615,257	10,332,971	13,262,416	9,910,536	12,789,688

Annual revenue of wind farm with and without CDM revenue which is calculated by SBM, DA, SQP and PDM is shown in Figs. 4, 5 and Table 3. As can be seen from the above figure and table, when the carbon equivalent transaction price set at 16.4$/t, the annual profit of a wind farm with CDM projects is about 3 million US dollars more than that of a wind farm without CDM projects. Therefore, when uncertainties such as electricity prices decline, the CDM revenue of wind farms can play a positive role in compensation for the annual revenue. By using the split Bregman method, the total annual revenue of wind farms is the largest and the value of it will change slightly with the change of the given value of reactive power $${Q}_{totalref}$$. When the given reactive power is plus or minus 0.3 p.u., supported by a CDM project, the AR calculated by the SBM algorithm increases by 1.87% and 1.23% respectively compared with the DA algorithm, the AR calculated by the SBM algorithm increases by 5.68% and 3.92% respectively compared with the SQP algorithm. The AR calculated using the SBM algorithm increased by 8.99% and 7.76% respectively compared with the PDM algorithm. When the given reactive power is plus or minus 0.2 p.u., the AR calculated by the SBM algorithm under the conditions supported by the CDM project increases by 0.93% and 0.52% respectively compared with the benefit calculated by the DA algorithm. The AR calculated by the SBM algorithm under the conditions supported by the CDM project increases by 2.37% and 1.48% respectively compared with the benefit calculated by the SQP algorithm. The AR calculated using the SBM algorithm increased by 4% and 3.32% respectively compared with the AR calculated by the PDM algorithm. When the given reactive power value is 0 p.u, wind farms with CDM projects have an annual revenue of about $13.84 million calculated by SBM, while without CDM projects, the annual revenue calculated by SBM is about $10.85 million.

It can be clearly seen from Figs. 6, 7, 8, 9, and 10, the total loss of wind farm is depends on the total reactive power given value of the system, the total reactive power given value is mutually exclusive with the power generation of wind power. As shown in Fig. 6, when the given value of reactive power is 0.3 p.u, the loss of the whole wind farm is the largest and the energy production is smallest. As can be seen from Fig. 6a, even in the place where the power calculated by the four algorithms are most similar (1005–1095 s), it is still clear that the power calculated by SBM and DA is significantly higher than that of SQP and PDM, thus demonstrating the advantages of distributed algorithm. From the loss diagram in Fig. 6b, the loss of SBM is close to that of DA, but the loss of SBM is still lower than that of DA. At this time the income calculated by SBM algorithm without CDM revenue is $10,783,278, which is an increase of 2.14% compared to the DA algorithm, an increase of 6.54% compared to the SQP algorithm and 10.39% compared to the PDM. The income with CDM revenue is $13,766,335, which is an increase of 1.87% compared with the DA, 5.68% compared with the SQP and 8.99% compared to the PDM.

As shown in Fig. 7a, when the given value of the reactive power is −0.3 p.u, the energy production calculated by the DA algorithm is similar to the energy production calculated by the SBM algorithm, but it can still be seen that the energy production value calculated by the SBM algorithm is slightly higher. In Fig. 7b, when the given value of the reactive power is −0.3 p.u, the difference between the losses of SQP and PDM is uniform, while the loss difference between SBM and DA algorithm is more similar. When the absolute value of ${Q}_{totalref}$ is the same, the total loss is higher when the value of reactive power ${Q}_{totalref}$ is positive than the value of reactive power ${Q}_{totalref}$ is negative. The reason is that the loss in a wind turbine is minimal when they absorb a certain amount of reactive power for excitation^26^.

When the given value of reactive power is 0.2 p.u, as shown in Fig. 8a, the total energy production is slightly larger than when the reactive power set value is plus or minus 0.3 p.u. Although the difference between the four algorithms is relatively small, it can still be seen from Figure that the power generation calculated by SBM is the largest. Compared with Fig. 6b, Fig. 8b has a relatively small loss of less than 0.02 p.u at 0 to 250 s, and then fluctuates between 0.01 p.u and 0.03 p.u at 250 s to 1000 s, finally reaches 0.07 p.u to 0.09 p.u as the power generation increases after more than 1000 s. The income calculated by SBM without CDM revenue is $10,822,473 in this situation, which is an increase of 1.06% compared to the DA, 2.71% compared to the SQP and 4.59% compared to the PDM. The income with CDM revenue is $13,810,196, which is an increase of 0.93% compared to the DA, 2.37% compared with the SQP algorithm and 4.01% compared to the PDM.

As can be seen in Fig. 9a, when the given value of reactive power is 0 p.u, there is almost no difference in the power generation calculated by all algorithms. However, it can still be seen from Fig. 9b that the loss calculated by PDM is larger than that calculated by DA, SQP and SBM. When the given value of reactive power is 0 p.u, the loss of the whole wind farm is the least and the return of wind farm is the greatest, the greater the absolute value of given reactive power, the greater the network loss of the system and the smaller the power generation. This is because the larger the reactive power rating of the system, the smaller the available active power that the system can provide, and the smaller the total power generation capacity. In order to discuss the influence of control parameters, Fig. 10a–d show the primal residual values of the SBM when the penalty factors are 1, 0.1, 0.05 and 0.005 respectively. Due to the large amount of data and variables, only the reactive power flows of the first four WT buses at the 1st, 2st, 3st and 4st feeder are shown here. When the penalty factor increases from 0.005 to 1, the convergence speed of the algorithm is accelerated, and the primal residual of 100 iterations can be reduced from ${10}^{-5}$ to ${10}^{-12}$. However, at the same time, the oscillation and fluctuation caused by rapid convergence also increase. Therefore, in order to avoid the oscillation and fluctuation caused by the large penalty factor, we chose the penalty factor of 0.005 to make the convergence more gentle and stable in this paper. Figure 10 (e) present the primal residual value of DA algorithm. By comparing Fig. 10d,e, the algorithm used in this paper can achieve convergence in 50 times, while DA algorithm needs 100 times to achieve convergence.

## Conclusion

A distributed ORPD strategy with the SBM is proposed in this paper. Compared with the continuation method, the value of penalty factor in SBM remains constant. And an appropriate value of the penalty factor can minimize the number of the condition of sub-problems, which contributes the fast convergence for iterative optimization methods and easily parallelization. Compared with the DA method, the SBM has a better scalability due to less dependence on parameters. Meanwhile, it takes the SBM used in this paper only ten seconds to iterate one hundred times in a single discrete time point, while the DA algorithm needs eighteen seconds. Compared with the centralized optimal control strategy SQP and PDM, the proposed method can reduce the interference information of signal transmission. Each node has its own set of constraints and its own objective function, each controller can complete its own function independently. By modularizing the system, the reusability of modules can be improved, the expansibility of the system is also higher. The power balance equality constraints, the voltage constraints and the reactive power limit of each wind turbine are considered by the proposed optimization method. A financial model of annual revenue is conducted to study the income impact with and without certified emission reduction (CER) by the CDM. Compared with the DA, SQP and the PDM, the annual revenue (AR) of the wind farm is the highest by using the SBM. When the given value of reactive power is 0.3 p.u, the AR calculated by SBM with CDM revenue is an increase of 0.93% compared to the DA, 2.37% compared with the SQP algorithm and 4.01% compared to the PDM. The AR calculated by SBM without CDM revenue is $10,783,278, which is an increase of 2.14% compared to the DA algorithm, an increase of 6.54% compared to the SQP algorithm and 10.39% compared to the PDM. Simulation results show the superiority of the proposed strategy. Furthermore, future work will focus on the exploration of multi-objective optimization. And it is meaningful to use the proposed strategy to develop cooperation strategies for the reactive and active power to improve the operation of wind farm furtherly.

## Supplementary Information

Supplementary Information.

## Data Availability

The data that support the fndings of this study are available from the corresponding authors Lingqi He and Mingcheng Lyu on reasonable request.
