# Supplementary material for: A split Bregman method solving optimal reactive power dispatch for a doubly-fed induction generator-based wind farm
Source: Sci Rep. 2022 Nov 10;12:19222. doi: 10.1038/s41598-022-17761-4 (PMC9649809; doi:10.1038/s41598-022-17761-4)
Supplement: Supplementary file 1 — Supplementary Information. [file 41598_2022_17761_MOESM1_ESM.docx]

**A Split Bregman method solving optimal reactive power dispatch for a doubly fed induction generator-based wind farm**

Fei Rong^1^, Lingqi He^1*^, Sheng Huang^1^, Mingcheng Lyu^1*^, Chao He^2^, Xueping Li^1^, Chunyi Zhao^3^

^1^College of Electrical and Information Engineering, Hunan University, Changsha 410082, China

^2^College of Automation Engineering, Nanjing University of Aeronautics and Astronautics, Nanjing 211106, China

^3^Hunan Shuangpai Hydropower Co., Ltd, Shuangpai 425200, China

Lingqi He ([hlq@hnu.edu,cn](mailto:hlq@hnu.edu,cn)) and Mingcheng Lyu ([289763620@qq.com](mailto:289763620@qq.com)) are the co-corresponding author.

**Appendix**

Here we give a detailed proof of the convergence of the split Bregman algorithm. Aim to

|  | $\min_{u}\mathcal{\vert D}u\vert,\text{ subject to }Au=f\text{.}$ | **(49)** |
| --- | --- | --- |

The problem can be transformed into

|  | $\min_{u} \left\vert\mathcal{D}u \right\vert+H\left( u \right)$ | **(50)** |
| --- | --- | --- |

Where $H(u)=\frac{\lambda}{2}\parallel Au-f\parallel^{2}$, we replaces the term |*Du*| in (49) and (50) by a separable one |*d*|, and then adds a new constraint *d = Du* into (49) and (50). Hence, (50) becomes

|  | $\min_{u} \vert d\vert+H(u),\text{ subject to }d=\mathcal{D}u$ | **(51)** |
| --- | --- | --- |

Then use split Bregman iteration as follow:

|  | $\left\{ \begin{aligned} &u^{k+1}=arg\min_{u} H\left( u \right)+\frac{\lambda}{2}\left\Vert d^{k}-Du-b^{k} \right\Vert_{2}^{2}, \\ &d^{k+1}=\arg\min_{d} \vert d\vert+\frac{\lambda}{2}\left\Vert d-\mathcal{D}u^{k+1}-b^{k} \right\Vert_{2}^{2}, \\ &b^{k+1}=b^{k}+\left( \mathcal{D}u^{k+1}-d^{k+1} \right). \end{aligned} \right.$ | **(52)** |
| --- | --- | --- |

One theorem is given as follow first:

THEOREM 1. Assume that there exists at least one solution $u^{*}$ of (50). Assume that $\lambda>0$. Then, we have the following properties for the split Bregman iteration (52):

|  | $\lim_{k\to+\infty} \left\vert\mathcal{D}u^{k} \right\vert+H\left( u^{k} \right)=\left\vert\mathcal{D}u^{*} \right\vert+H\left( u^{*} \right)$ | **(53)** |
| --- | --- | --- |

Furthermore,

|  | $\lim_{k\to+\infty} \left\Vert u^{k}-u^{*} \right\Vert=0$ | **(54)** |
| --- | --- | --- |

Whenever (50) has a unique solution.

Then we prove the theorem given above.
Proof. Let $u^{*}$ be an arbitrary solution of (50). By the first order optimality condition, $u^{*}$ must satisfy

|  | $0=\mathcal{D}^{T}p^{*}+\nabla H\left( u^{*} \right)$ | **(55)** |
| --- | --- | --- |

Where $p^{*}\in\partial\left| d^{*} \right|$ with $d^{*}=\mathcal{D}u^{*}$. Let

|  | $b^{*}=\frac{1}{\lambda}p^{*}$ | **(56)** |
| --- | --- | --- |

We obtain

|  | $\left\{ \begin{matrix} 0=\nabla H\left( u^{*} \right)+\lambda\mathcal{D}^{T}\left( d^{*}\mathcal{-D}u^{*}-b^{*} \right), \\ 0=p^{*}+\lambda\left( d^{*}-\mathcal{D}u^{*}-b^{*} \right), \text{with }p^{*}\in\partial\left\vert d^{*} \right\vert, \\ b^{*}=b^{*}+\left( \mathcal{D}u^{*}-d^{*} \right). \end{matrix} \right.$ | **(57)** |
| --- | --- | --- |

Therefore, $u^{*},d^{*},b^{*}$ is a fixed point of the split Bregman iteration (52). Consequently, if the split Bregman iteration converges, it converges to a solution of (50).

Denote the errors by

|  | $u_{e}^{k}=u^{k}-u^{*}, d_{e}^{k}=d^{k}-d^{*}, b_{e}^{k}=b^{k}-b^{*}.$ | **(58)** |
| --- | --- | --- |

Then

$$\frac{\lambda}{2\delta}\left( \left\| b_{e}^{0} \right\|^{2}-\left\| b_{e}^{K+1} \right\|^{2} \right)$$

$$= \sum_{k=0}^{K} \left\langle\nabla H\left( u^{k+1} \right)-\nabla H\left( u^{*} \right),u^{k+1}-u^{*} \right\rangle+\sum_{k=0}^{K} \left( p^{k+1}-p^{*},d^{k+1}-d^{*} \right\rangle$$

$+\lambda\left( \frac{1-\delta}{2}\sum_{k=0}^{K} \left\| \mathcal{D}u_{e}^{k+1}-d_{e}^{k+1} \right\|^{2}+\frac{1}{2}\sum_{k=0}^{K} \left\| \mathcal{D}u_{e}^{k+1}-d_{e}^{k} \right\|^{2}+\frac{1}{2}\left\| d_{e}^{K+1} \right\|^{2} \right)-\frac{\lambda}{2}\left\| d_{e}^{0} \right\|^{2}$ (59)

Note that all terms involved in the above equation are nonnegative. This observation leads to the following inequality:

|  | $\frac{\lambda}{2\delta}\left\Vert b_{e}^{0} \right\Vert^{2}+\frac{\lambda}{2}\left\Vert d_{e}^{0} \right\Vert^{2}\geq\sum_{k=0}^{K} \left\langle\nabla H\left( u^{k+1} \right)-\nabla H\left( u^{*} \right),u^{k+1}-u^{*} \right\rangle.$ | **(60)** |
| --- | --- | --- |

By assumption $\lambda>0$, we have $\sum_{k=0}^{+\infty} \left\langle\nabla H\left( u^{k+1} \right)-\nabla H\left( u^{*} \right),u^{k+1}-u^{*} \right\rangle<+\infty$, which leads to

|  | $\lim_{k\to+\infty} \left\langle\nabla H\left( u^{k} \right)-\nabla H\left( u^{*} \right),u^{k}-u^{*} \right\rangle=0.$ | **(61)** |
| --- | --- | --- |

This, together with the nonnegativity of the Bregman distance, implies that $\lim_{k\to+\infty} B_{H}^{\nabla H\left( u^{*} \right)}\left( u^{k},u^{*} \right)=0$, i.e.,

|  | $\lim_{k\to+\infty} H\left( u^{k} \right)-H\left( u^{*} \right)-\left\langle u^{k}-u^{*},\nabla H\left( u^{*} \right) \right\rangle=0.$ | **(62)** |
| --- | --- | --- |

Similarly, we can prove that $\lim_{k\to+\infty} B_{|-|}^{p^{*}}\left( d^{k},d^{*} \right)=0$, i.e.,

|  | $\lim_{k\to+\infty} \left\vert d^{k} \right\vert-\left\vert d^{*} \right\vert-\left\langle d^{k}-d^{*},p^{*} \right\rangle=0$ | **(63)** |
| --- | --- | --- |

And

|  | $\lim_{k\to+\infty} \left\Vert\mathcal{D}u^{k+1}-d^{k} \right\Vert=0$ | **(64)** |
| --- | --- | --- |

Since $|\cdot|$ is continuous, by (63) and (64), we obtain

|  | $\lim_{k\to+\infty} \left\vert\mathcal{D}u^{k} \right\vert-\left\vert\mathcal{D}u^{*} \right\vert-\left\langle\mathcal{D}u^{k}-\mathcal{D}u^{*},p^{*} \right\rangle=0$ | **(65)** |
| --- | --- | --- |

Summing this and (62) up, it yields

|  | $\lim_{k\to+\infty} \left( \left\vert\mathcal{D}u^{k} \right\vert+H\left( u^{k} \right) \right)-\left( \left\vert\mathcal{D}u^{*} \right\vert+H\left( u^{*} \right) \right)-\left\langle u^{k}-u^{*},\nabla H\left( u^{*} \right)+\mathcal{D}^{T}p^{*} \right\rangle=0$ | **(66)** |
| --- | --- | --- |

This together with (55) proves (53). Next, we prove (54) by assuming that (50) has the unique solution $u^{*}$. It is proven by contradiction. Let $E(u)=|\mathcal{D}u|+H(u)$. Then $E(u)$ is a convex, lower semicontinuous function. Assume that $|\cdot|$ is the $\mathcal{l}_{1}$ norm and $\mathcal{D}$ has a left inverse (e.g., $\mathcal{D}$ is a tight frame). This theorem implies that the energy $\left| \mathcal{D}u^{k} \right|+H\left( u^{k} \right)$ of $u^{k}$ converges to the minimum value of $|\mathcal{D}u|+H(u)$. In other words, the split Bregman algorithm (52) reaches a solution that has an energy arbitrarily close to the minimum energy with sufficient number of iterations. This shows that one can get the right solution numerically by (52). Furthermore, $u^{k}$ has a convergent subsequence, as $\left| \mathcal{D}u^{k} \right|$ and $\left\| u^{k} \right\|$ are bounded. Finally, when $H(u)=\parallel Au-f\parallel^{2}$, which is commonly used in many applications, and when $A$ is invertible, $u^{k}$ converges to the unique solution of (50).
